# Unveiling Tick Diversity in Cattle in Cameroon: Emergence of *Rhipicephalus microplus*, Replacing the Original *Rhipicephalus* spp.

**DOI:** 10.3390/vetsci12020123

**Published:** 2025-02-03

**Authors:** Muhammad Umair Aziz, Jehan Zeb, Michel Lontsi-Demano, Angel Almendros, José de la Fuente, Olivier Andre Sparagano, Patrick Butaye

**Affiliations:** 1 Department of Infectious Diseases and Public Health, Jockey Club College of Veterinary Medicine and Life Sciences, City University of Hong Kong, Hong Kong 999077, China; muhamaziz3-c@my.cityu.edu.hk (M.U.A.); olivier.sparagano@ukmc.ac.uk (O.A.S.); 2 Center for Immunology and Infection Limited, Hong Kong Science and Technology Park, Hong Kong, China; jehan.zeb@c2i.hk; 3 International Institute of Tropical Agriculture (IITA), Cotonou P.O. Box 0932, Benin; michel.lontsi@yahoo.fr; 4 Department of Veterinary Clinical Sciences, Jockey Club College of Veterinary Medicine and Life Sciences, City University of Hong Kong, Kowloon, Hong Kong, China; aalmendr@cityu.edu.hk; 5 Health and Biotechnology (SaBio), Instituto de Investigación en Recursos Cinegéticos (IREC)-Spanish National Research Council (CSIC)-University of Castille La Mancha (UCLM)-Castilla La Mancha Regional Council (JCCM), Ronda de Toldo 12, 13005 Ciudad Real, Spain; jose_delafuente@yahoo.com; 6 Department of Veterinary Pathobiology, Center for Veterinary Health Sciences, Oklahoma State University, Stillwater, OK 74078, USA; 7 UK Management College, Manchester M11 1AA, UK; 8 Department of Pathobiology, Pharmacology and Zoological Medicine, Faculty of Veterinary Medicine, Ghent University, 9820 Merelbeke, Belgium

**Keywords:** ticks, tick-borne diseases, *cox1*, *16s*, *Rhipicephalus microplus*, Cameroon

## Abstract

Ticks are well-known vectors of pathogens, posing considerable risks to livestock health and productivity. In Cameroon, where livestock production is vital, established tick species such as *Amblyomma variegatum* and *Hyalomma truncatum* are prevalent in key cattle-rearing areas. The recent introduction of the invasive, acaricide-resistant *Rhipicephalus microplus* further complicates control efforts. In this study, we deliver baseline data on the composition of tick species and their shifts within agroecological zone I (AEZ I) and agroecological zone III (AEZ III) of Cameroon, providing a foundation for targeted vector control strategies to ultimately reduce the risk of pathogen transmission. From 1100 ticks collected across two Cameroonian agroecological zones, *H. truncatum* (39.9%), *A. variegatum* (31%), and *R. microplus* (10.64%) were the most prevalent species, along with *Rhipicephalus lunulatus* (4.45%), *Hyalomma rufipes* (1.45%), *Hyalomma marginatum* (1.09%), *Hyalomma dromedarii* (0.45%), and *Rhipicephalus sanguineus* (0.9%). Molecular identification using cytochrome C oxidase subunit 1 (*cox1)* and *16s* led to the identification of five additional species, *Hyalomma nitidum* (0.73%), *Rhipicephalus simus* (3.54%), *Rhipicephalus sulcatus* (2.64%), *Rhipicephalus praetextatus* (2.1%), and *Rhipicephalus pusillus* (1.1%). *R. microplus* emerged as the most dominant *Rhipicephalus* spp. in AEZ I, comprising 67.5% of the total *Rhipicephalus* ticks. In conclusion, we demonstrate the further spread of *R. microplus*, which represents a major source of vector-borne diseases, affecting both humans and animals.

## Simple Summary

Ticks are a major threat to livestock health and productivity, especially in Cameroon, where cattle farming is vital. In this study, we examined ticks from two key cattle-rearing areas in Cameroon, agroecological zone I (AEZ I) and zone III (AEZ III). A total of 1100 ticks were collected, revealing that the most common species were *Hyalomma truncatum* (39.9%), *Amblyomma variegatum* (31%), and the invasive, acaricide-resistant *Rhipicephalus microplus* (10.64%). The invasive *R. microplus* is outcompeting native ticks and serves as a major vector for diseases significant to both animal and public health. This research provides essential data on tick species and their distribution, emphasizing the urgent need for more effective strategies to control *R. microplus* and protect livestock in Cameroon and western Central Africa.

## 1. Introduction

Ticks and tick-borne diseases (TBDs) are estimated to cause substantial economic losses of around USD 20–30 billion annually worldwide. Specifically, Eastern Africa is experiencing annual losses of approximately USD 364 million [1,2]. Ticks and TBDs are significant threats to livestock production in sub-Saharan Africa and public health [3]. This threat is expected to increase due to climate change and the increased movement of livestock [4]. The heterogeneity of livestock production systems, ranging from semi-intensive small-scale operations in highlands to extensive pastoral systems in arid regions, complicates the understanding of TBD dynamics across the continent. Cameroon, a country in Central Africa, through the transhumance of livestock and livestock trade, epidemiologically connects West Africa and beyond [5], promoting the spread of ticks and TBDs. Recently, the invasive cattle tick *Rhipicephalus microplus*, a key vector of *Babesia bovis*, *Anaplasma marginale,* and *Babesia bigemina* was introduced to West Africa as a result of cattle imports from Brazil and has since quickly expanded its range [6,7]. This tick is notorious for its invasive nature and acaricide resistance, which pose a growing threat to livestock productivity and health across the region [8,9]. *Rhipicephalus microplus* and other ticks, like *R. simus* and *R. praetextatus*, are becoming increasingly common, spreading *A. marginale*, *A. centrale*, *B. bovis, B. bigemina, Theileria annulata*, *Rickettsia conorii*, and the Nairobi sheep disease virus [10,11,12,13]. While *Amblyomma variegatum* and *Hyalomma truncatum* remain predominant in the region, the emergence and potential range expansion of new *Rhipicephalus* species underscores the dynamic nature of tick communities and their evolving threat to livestock [14].

Tick surveillance in Cameroon has predominantly relied on morphological identification methods, which are not always able to accurately distinguish between closely related species, particularly when dealing with damaged, engorged, or immature specimens [15]. However, the accurate identification of tick species is pivotal for effective surveillance and control measures. Therefore, we conducted a morphological and molecular survey of tick species infesting cattle in Cameroon’s major livestock production zones, agroecological zone 1 (AEZ I) and the Western Highlands, also known as agroecological zone 3 (AEZ III), which account for nearly 66% of the cattle population. The aim was to assess whether *R. microplus* and other *Rhipicephalus* species, alongside *Amblyomma* and *Hyalomma*, are expanding their range.

## 2. Methodology

### 2.1. Sampling Collection

**Figure 1:**
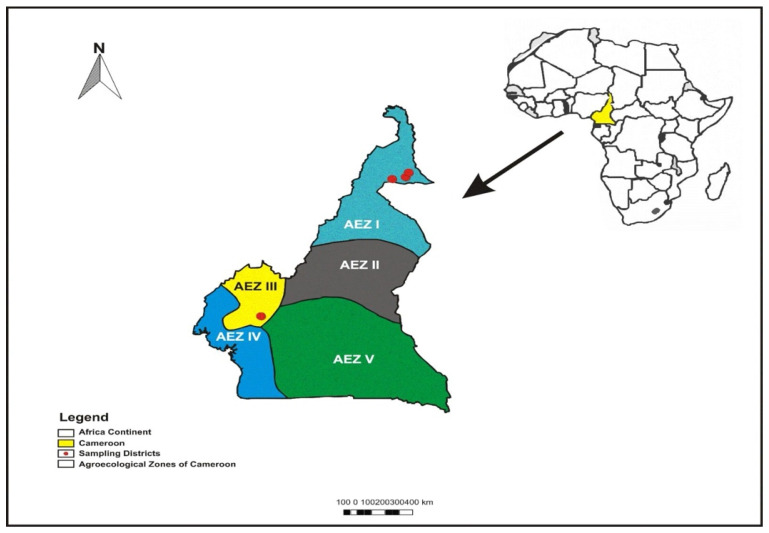
Map highlighting the study districts from two different agroecological zones (AEZs) of Cameroon.

This study was conducted as a cross-sectional survey to assess tick species distribution and infestation rates among cattle in Cameroon. The survey was carried out in two agroecological zones (AEZs): the North and Far North (AEZ I) and the Western Highlands (AEZ III), during their respective peak rainy seasons in 2022 (Figure 1). AEZ I, the Sudano-Sahelian zone, has high temperatures and dry savannah and steppes. Around 1.89 million heads of cattle are found there [16]. AEZ III (Western Highlands) features mountainous terrain, lower temperatures, and high rainfall, supporting an estimated cattle population of 1.98 million heads [17].

Cattle were sampled from medium-sized farms (50–200 animals) across selected districts: Kalfou, Touloum, and Kaélé in the Far North and Bangangté in the Western Highlands. The number of farms visited was based on cattle density and the farmers’ willingness to participate in the survey. Stratified random sampling was employed, with 10–15% of animals per farm selected. The geographic coordinates of the study sites are as follows:

- **Far North (AEZ I)**: Kalfou (N 10° 2′ 4.92″, E 14° 26′ 3.84″), Touloum (N 10° 15′ 50.40″, E 14° 30′ 30.60″), and Kaélé (N 10° 6′ 34.56″, E 14° 27′ 2.88″).
- **Western Highlands (AEZ III)**: Bangangté (N 05° 8′ 24″, E 10° 31′ 12″).

Adult ticks were collected from cattle using blunt steel forceps and preserved in 70% ethanol at 4 °C. To ensure a representative sample of tick diversity, up to five ticks were collected per infested animal. The majority of the sampled cattle were local breeds, with a few crossbreed animals. They were mostly managed under open grazing systems, with occasional combined stall-feeding practices.

### 2.2. Morphological Identification of Ticks

Ticks were identified morphologically exercising standard taxonomic keys under a stereomicroscope at 100× magnification [13].

### 2.3. DNA Extraction and PCR

Of each morphologically identified tick species, a representative subset (20%) from each genus was selected for DNA extraction and PCR. Ticks were dissected into small pieces using sterile scalpels. The Qiagen DNeasy Blood and Tissue Kit (Qiagen, Hilden, Germany) was used for DNA extraction and the extracted DNA was stored at −20 °C until it was used for PCR amplification. Nanodrop (Thermo Fisher Scientific, Waltham, MA, USA) was used to measure the DNA concentration.

PCR reactions for mitochondrial 16s ribosomal RNA (*16s rRNA*) and *cox1* were performed as described before [18]. The primers used in this study target conserved regions that are applicable across multiple genera of ticks, allowing for broad amplification and subsequent identification. Briefly, a total volume of 30 µL was used, containing 2.5 µL 100 ng genomic DNA, 10.5 µL of nuclease-free water (Thermo Fisher Scientific, Waltham, MA, USA), 1 µL of each primer (10 pmol), and 15 µL of DreamTaq PCR master mix (2X) (Thermo Fisher Scientific, Waltham, MA, USA). ProFlex thermocycler (Thermo Fisher Scientific, Waltham, MA, USA) was used for PCR. For the *16s* rRNA gene, the thermal cycling conditions were an initial denaturation at 95 °C for 4 min, followed by 35 cycles of denaturation at 95 °C for 1 min, annealing at 50 °C for 1 min, and extension at 68 °C for 1 min. This was followed by a final extension at 68 °C for 10 min. The following primers were used: Forward: 16S + 1 (5′-CTGCTCAATGATTTTTTAAATTGCTGTGG-3′) and Reverse: 16S-1 (5′-CCGGTCTGAACTCAGATCAAGT-3′). The size of the amplified fragment was 460 base pairs [18]. For the *cox1* gene, the conditions were as follows: initial denaturation at 95 °C for 5 min, followed by 40 cycles of 95 °C for 45 s, annealing at 50 °C for 45 s, and extension at 72 °C for 1 min. This was followed by a final extension at 72 °C for 5 min. The forward and reverse primer sequences used for amplifying the *cox1* gene are as follows: Forward: LCO1490 (5′- GGTCAACAAATCATAAAGATATTGG-3′) and Reverse: HCO2198 (5′-TAAACTTCAGGGTGACCAAAAAATCA-3′). The size of the amplified fragment was 710 base pairs [19].

PCR products were analyzed on 2% agarose gels, stained with SYBR Safe dye (Invitrogen Thermo Fisher, Waltham, MA, USA), and visualized under UV light using a Gel doc EZ gel documentation system (Bio-Rad, Hercules, CA, USA).

### 2.4. Sequencing and Sequence Analysis

PCR products were sent for Sanger sequencing to BGI genomics (Hong Kong, China). The resulting sequences were edited and trimmed using 4Peaks (v1.8) software. The sequences were compared in the GenBank database using the BLASTn program for identification of the ticks [20]. An identity percentage above 97% in BLAST analysis with GenBank sequences is considered the threshold for tick species identification.

### 2.5. Phylogenetic Analysis

Multiple sequence alignments were performed using MUSCLE in MEGA11 (version 11.0.13) [21] and phylogenetic analyses were conducted using the maximum likelihood (ML) method implemented in IQ-TREE 2 (v2.3.6) [22]. IQ-TREE’s ModelFinder module was used to automatically select the best-fit substitution model based on the Bayesian Information Criterion (BIC). ML analyses were conducted with 1000 ultrafast bootstrap replicates to estimate branch support, ensuring a robust evaluation of the inferred phylogeny. Separate trees were generated based on both *cox1* and *16s* genes. Final visualization and editing were performed by interactive tree of life (iTOL v7) [22].

### 2.6. Statistical Analysis

Data were compiled using MS Excel, and the analysis was performed in R version 4.3.1. A chi-square test was employed to examine the relationship between different study locations and tick species. A statistical significance level of 95% was established, with a *p*-value of less than 0.05 considered statistically significant.

## 3. Results

### 3.1. Tick Collection and Identification

**Figure 2:**
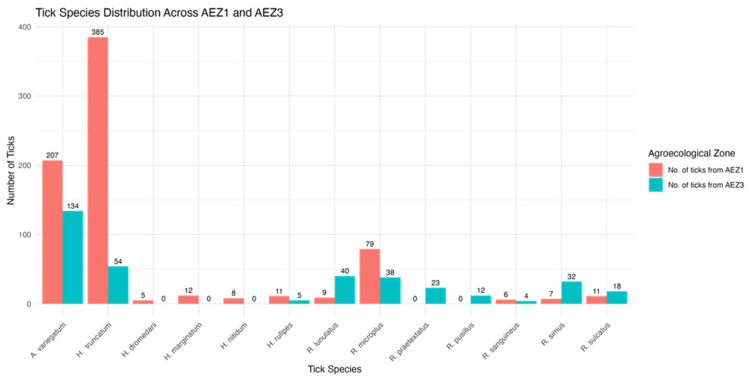
Tick distribution across both agroecological zones.

A total of 1100 ticks were collected from 515 cattle across fifteen sites located in four districts, covering two agroecological zones (AEZs). Ticks were collected during the rainy season. Morphological identification revealed three genera: *Amblyomma* (341, 31%,), *Hyalomma* (480, 43.64%), and *Rhipicephalus* (250, 22.73%) (Table 1). A bar plot illustrating the detailed distribution of each tick species across both agroecological zones (AEZs) is presented (Figure 2). There were statistically significant differences (*p*-value < 0.05) between agroecological zones and various tick genera within AEZ I and AEZ III.

A total of 210 samples from the microscopically identified ticks, a representative subset (20%), were randomly selected for identification using PCR and sequencing. A total of 13 different species were found: *A. variegatum* (341, 31%), *H. truncatum* (439, 39.9%), *H. rufipes* (16, 1.45%), *H. marginatum* (12, 1.09%), *H. nitidum* (8, 0.73%), *H. dromedarii* (5, 0.45%), *R. microplus* (117, 10.64%), *R. lunulatus* (49, 4.45%), *R. simus* (39, 3.54%), *R. sulcatus* (29, 2.64%), *R. praetextatus* (23, 2.1%), *R. pusillus* (12, 1.1%), and *R. sanguineus* (10, 0.9%). The identification corresponded to the morphological identification.

Of the collected ticks, 53 samples were classified only to the genus level based on morphology (17 from the genus *Hyalomma* and 36 from the genus *Rhipicephalus*). These samples were further identified at the species level using PCR and sequencing. The 17 samples of *Hyalomma* consisted of 10 *H. truncatum*, 3 *H. rufipes*, 2 *H. marginatum*, 1 *H. dromedarii*, and 1 *H. nitidum*. The 36 samples of *Rhipicephalus* were identified as 6 *R. microplus*, 5 *R. sulcatus*, 4 *R. praetextatus*, 7 *R. simus*, 11 *R. lunulatus*, 2 *R. pusillus*, and 1 *R. sanguineus*.

*Rhipicephalus microplus* was the dominant *Rhipicephalus* species in AEZ I, representing 67.52% (n = 79) of the *Rhipicephalus* spp. The remaining six *Rhipicephalus* species were *R. lunulatus*, *R. simus*, *R. sulcatus*, *R. praetextatus*, *R. pusillus*, and *R. sanguineus*, who represented 20.38% (n = 33) of the total *Rhipicephalus* spp. Within the districts of Kaélé and Kalfou, *R. microplus* prevalence was 47.01% (n = 55) and 15.38% (n = 18) of collected ticks, respectively, with no *R. microplus* observed in Touloum. In AEZ III, which is represented by the district Bangangté, *R. microplus* comprised 32.48% (n = 38) of the *Rhipicephalus* spp. collected, with other *Rhipicephalus* species contributing 79.62% (n = 129) (Table 2).

### 3.2. Phylogenetic Analysis of the 16s and cox1 Gene:

**Figure 3:**
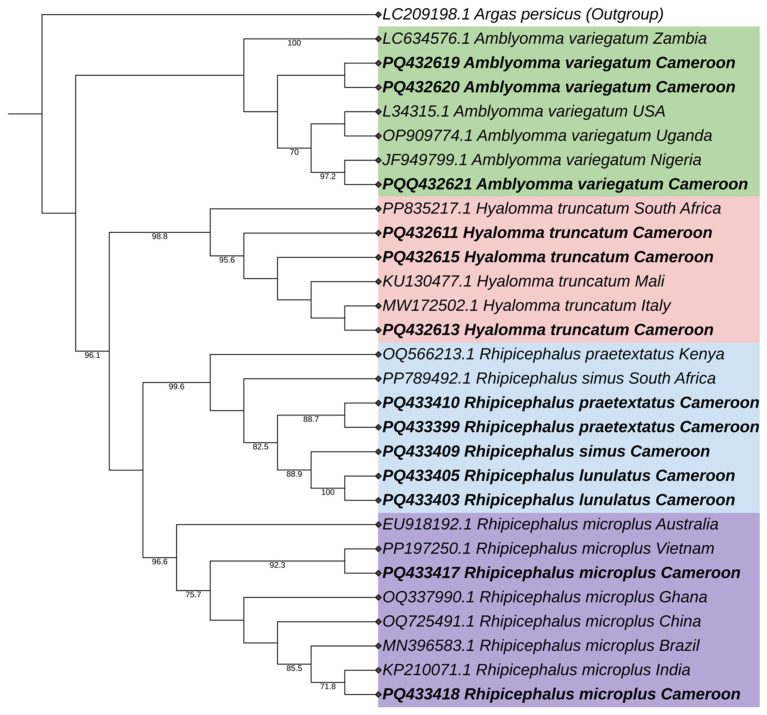
Maximum likelihood (ML) phylogenetic tree of the *Amblyomma*, *Hyalomma*, and *Rhipicephalus* tick genus based on *16s* ribosomal gene marker. Sequences obtained from this study are highlighted in bold, and different clades are distinguished using color highlights. LC209198.1 (*Argas persicus*) was used as the outgroup.

A total of thirteen partial *16s* sequences representing all identified tick species were included in this study, complemented by sixteen global reference *16s* sequences sourced from the GenBank database. These sequences were utilized to infer the phylogenetic relationships among various taxa using the maximum likelihood method (Figure 3). The resulting *16s* phylogenetic tree revealed four distinct clades, each demonstrating strong bootstrap values (exceeding 70%) at the majority of nodes.

The first clade consists of members of *A. variegatum*. The second clade comprises sequences from the *H. truncatum* species. The third clade is formed by the members of the ‘*Rhipicephalus simus* group’, exhibiting high bootstrap support above 99%. This group includes sequences from *R. simus*, *R. praetextatus*, and *R. lunulatus*. Finally, *R. microplus* is represented in a separate clade.

**Figure 4:**
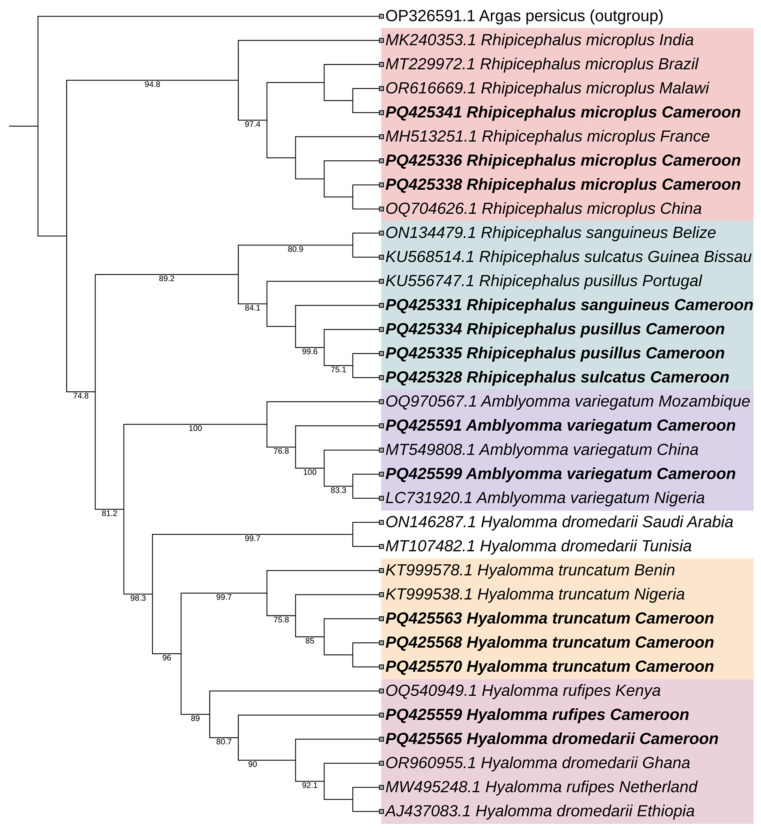
Maximum likelihood (ML) phylogenetic tree of the *Amblyomma, Hyalomma,* and *Rhipicephalus* tick genus based on *cox1* gene marker. Sequences obtained from this study are highlighted in bold, and different clades are distinguished using color highlights. OP326591.1 (*Argas persicus*) was used as the outgroup.

Based on partial *cox1* sequences, a second phylogenetic tree was constructed, incorporating twenty reference sequences from the GenBank database worldwide, along with fourteen sequences generated from this study, utilizing the maximum likelihood method (Figure 4). The analysis resulted in the formation of six distinct clades. The first clade includes sequences from the tick species *R. microplus*. The second clade comprises the ‘*Rhipicephalus sanguineus* group’, which includes *R. sanguineus*, *R. pusillus*, and *R. sulcatus*. *A. variegatum* clustered closely together to form a third clade. The members of *H. dromedarii* were divided into two separate groups; sequences from Saudi Arabia and Tunisia formed a small, distinct clade, while sequences from Cameroon, Ghana, and Ethiopia clustered separately alongside the *H. rufipes* tick species. *Hyalomma truncatum* formed its own distinct clade containing members of the same species.

## 4. Discussion

Effective surveillance of various tick species and their pathogens is critical for alleviating the impact of tick-borne diseases (TBDs). Recently, the invasive tick *R. microplus*, which is resistant to acaricides, has made its way into West Africa [7,8,23]. This development has created significant challenges in managing tick infestations in cattle. Furthermore, the cattle trade in this region is not controlled, creating risks of disease dissemination [5]. Notorious for its adaptability and capability to outcompete native species, *R. microplus* presents a growing threat to animal health [7,24]. If its spread continues uncontrolled, it could significantly increase the economic burden on the livestock sector. Current surveillance data are limited, restricting our understanding of species distribution and hindering the development of effective control strategies.

Morphological identification, using sequencing and analyzing the sequences using BLAST, is a widely used method for the identification of ticks; however, it has limitations, particularly with species within the *Hyalomma* and *Rhipicephalus* genera, which often display overlapping characteristics. Previous research has underscored the necessity of employing molecular techniques to achieve accurate species identification when morphological analysis falls short [25,26]. In our study, molecular methods proved crucial for precisely identifying certain *Hyalomma* and *Rhipicephalus* species. This included *R. sulcatus* from *R. praetextatus*, *R. lunulatus*, *R. pusillus*, *R. sanguineus*, *R. simus*, as well as *H. truncatum*, *H. rufipes*, *H. marginatum*, *H. dromedarii*, and *H. nitidum*, which could not be reliably distinguished based solely on morphological traits.

Identifying *Rhipicephalus* species using phylogenetic assessment of the sequences poses challenges due to their high genetic similarity [27]. Phylogenetic analysis consistently placed *R. microplus* in distinct clades for both *cox1* and *16s*, allowing clear differentiation of this species. In contrast, based on the *16s* gene marker, *R. simus*, *R. praetextatus*, and *R. lunulatus* clustered in a single clade, reflecting close genetic ties within this group. Similarly, the phylogenetic tree based on *cox1* was unable to place *R. sanguineus*, *R. pusillus*, and *R. sulcatus* in a separate clade. This also highlights the limitations of the *cox1* and *16s* markers in distinguishing these species within this genus [28,29]. Moreover, the genes do not allow more in-depth molecular epidemiological studies. Thus, additional or new markers, such as ITS2, are necessary to enhance taxonomic resolution for closely related species in complex groups as described before [29] and epidemiological studies. Similarly in the genus *Hyalomma*, using phylogenetic analysis, *H. dromedarii* did not cluster in a separate species.

The difficulties encountered with species identification can be explained by introgressive hybridization, where one ’species’ incorporates genetic material from another ’species’ through ’interspecific hybridization’. Previous studies have demonstrated the discrepancy between morphological and molecular identification, which is believed to result from hybridization among *Hyalomma* taxa in Africa [26]. This difference can also be explained based on the markers’ resolution capacities: *16s*, a widely conserved gene for species identification, often lacks the sensitivity to capture fine-scale, within-species variation, while *cox1* can reveal regional or ecological sub-structuring, potentially highlighting adaptations to environmental conditions or geographic isolation [28,29,30,31,32].

Among the various tick species studied, *Hyalomma* species emerged as the most abundant, mainly in the AEZ I region, where *H. truncatum* was the predominant species. However, in AEZ III, *A. variegatum* was identified as the major tick. These results are consistent with previous studies [14,33,34], which also reported *H. truncatum* as the most prevalent species in AEZ I and *Amblyomma variegatum* as the key tick species in AEZ III. Along with that, *H. rufipes* is also detected, though to a limited extent, in both regions. The presence of *H. dromedarii*, recently documented in Northern Cameroon, has also been identified in this study within the northern districts of AEZ I albeit at low prevalence [3]. This region also has a large camel population and these ticks are probably only spill overs from camels. Furthermore, *A. variegatum* was consistently present across all districts in both regions, validating earlier findings documenting its widespread occurrence throughout the country [35,36].

The most noteworthy finding from this study is the emergence and expansion of *R. microplus* in AEZ I, where it has become the dominant tick vector among other *Rhipicephalus* species, successfully outcompeting the established native *Rhipicephalus* species [37,38]. *R. microplus* was previously primarily found in AEZ III and had not been reported in AEZ I [3]. Recent reports indicate that *R. microplus* is present at low prevalence within AEZ I [34]. The abrupt expansion of *R. microplus*, along with the apparent dislocation of native tick species, can likely be attributed to the greater egg production capacity and shorter life cycle of *R. microplus* [39]. This evolution has also recently been documented in South Africa, Ivory Coast, and Tanzania, both probably arising from separate introductions [40,41,42]. Additionally, its resistance to many existing acaricides may have contributed to its growth at the cost of susceptible species, who are eliminated from the acaricide treatments [43].

*Rhipicephalus microplus* is a known vector for several pathogens, including *B. bovis*, *A. marginale*, *B. bigemina*, and *Theileria equi*. Furthermore, the presence of *A. variegatum* and *H. truncatum*, which can transmit *Rickettsia* species, *Ehrlichia ruminantium*, and *Coxiella burnetii* (the causative agent of Q fever), as well as *Theileria annulata*, underscores the significant risk of tick-borne diseases (TBDs) in these regions. Longitudinal studies are needed to assess the permanence of these species and the factors driving *R. microplus* spread. Future research should utilize whole mitochondrial genome sequencing to improve species differentiation within the *Rhipicephalus* and *Hyalomma* genus. This method could enhance genetic data resolution, aiding in the identification of species and the development of targeted tick control strategies [44,45]. Given these findings, it is essential to implement control strategies targeting these local tick species and the pathogens they carry to reduce the disease burden in high-risk areas.

## 5. Conclusions

This study provides crucial data on the prevalence and diversity of tick species in Cameroon’s AEZ I and AEZ III. The increase in the occurrence of *R. microplus*, coupled with the risks posed by *A. variegatum* and *H. truncatum*, emphasizes the need for new localized tick control strategies. The invasive and acaricide-resistant *R. microplus* has rapidly established itself as the dominant species within its genus in AEZ I, continuing to expand into areas where it poses significant risks as a vector for zoonotic and animal diseases.

## Figures and Tables

**Table 1:** Distribution and percentage of tick species across agroecological zones and their districts.

Tick Genus	Tick Species	No. of Ticks Collected (%)	AEZ I District (%) Kaélé	District (%) Kalfou	District (%) Touloum	AEZ III Districts (%) Bangangté
*Amblyomma*	*A. variegatum*	341 (31)	102 (24.7)	57 (28.79)	48 (37.21)	134 (37.22)
*Hyalomma*	*H. truncatum*	439 (39.9)	196 (47.46)	114 (57.58)	75 (58.14)	54 (15)
	*H. rufipes*	16 (1.45)	11 (2.66)	0 (0)	0 (0)	5 (1.39)
	*H. marginatum*	12 (1.09)	7 (1.69)	5 (2.53)	0 (0)	0 (0)
	*H. nitidum*	8 (0.73)	8 (1.94)	0 (0)	0 (0)	0 (0)
	*H. dromedarii*	5 (0.45)	5 (1.21)	0 (0)	0 (0)	0 (0)
*Rhipicephalus*	*R. microplus*	117 (10.64)	55 (13.32)	18 (9.09)	6 (4.65)	38 (10.56)
	*R. lunulatus*	49 (4.45)	5 (1.21)	4 (2.02)	0 (0)	40 (11.11)
	*R. simus*	39 (3.54)	7 (1.69)	0 (0)	0 (0)	32 (8.89)
	*R. sulcatus*	29 (2.64)	11 (2.66)	0 (0)	0 (0)	18 (5)
	*R. praetextatus*	23 (2.1)	0 (0)	0 (0)	0 (0)	23 (6.39)
	*R. pusillus*	12 (1.1)	0 (0)	0 (0)	0 (0)	12 (3.33)
	*R. sanguineus*	10 (0.9)	6 (1.45)	0 (0)	0 (0)	4 (1.11)
	Total	1100	413	198	129	360

**Table 2:** Distribution % of *R. microplus* compared to other *Rhipicephalus* spp. per district.

Agroecological Zone	No. of Sites	No. of *R. microplus* (%)	No. of Other *Rhipicephalus* spp. (%)
AEZ I	12	79 (67.52)	33 (20.38)
Kaélé	6	55 (47.01)	29 (17.90)
Kalfou	4	18 (15.38)	4 (2.48)
Touloum	2	6 (5.13)	0 (0)
AEZ III	3	38 (32.48)	129 (79.62)
Bangangté	3	38 (32.48)	129 (79.62)

## Data Availability

Data are contained within the article.
